# Serum metabolite profiling reveals metabolic characteristics of sepsis patients using LC/MS-based metabolic profiles: a cross-sectional study

**DOI:** 10.1186/s12920-023-01666-w

**Published:** 2023-09-26

**Authors:** Jinliang Peng, Chongrong Qiu, Jun Zhang, Xiaoliu Xiao

**Affiliations:** Department of Emergency, The Affiliated Ganzhou Hospital of Nanchang university, Ganzhou, Jiangxi Province 341000 China

**Keywords:** Sepsis, Biomarkers, Metabonomic, Liquid chromatography-mass spectrometry, Phosphatidylcholines

## Abstract

**Background:**

Individuals with sepsis exhibited a higher likelihood of benefiting from early initiation of specialized treatment to enhance the prognosis of the condition. The objective of this study is to identify potential biomarkers of sepsis by means of serum metabolomics.

**Materials and methods:**

The screening of putative biomarkers of sepsis was conducted using serum samples from patients with sepsis and a control group of healthy individuals. The pathogenesis of sepsis was determined through the utilization of liquid chromatography-mass spectrometry-based metabolic profiles and bioinformatic techniques, which in turn provided a foundation for timely diagnosis and intervention.

**Results:**

Individuals with sepsis had significantly different metabolic characteristics compared to those with normal health. The concentrations of phosphatidylcholines (PCs), phosphatidylserine (PS), lysophosphatidylethanolamine (LysoPEs), and lysophosphatidylcholine (LysoPCs) exhibited a decrease, while the levels of creatinine, C17-Sphinganine, and PS(22:0/22:1(11Z)) demonstrated an increase in the serum of sepsis patients when compared to the control group. Additionally, ROC curves were generated to assess the discriminatory ability of the differentially expressed metabolites. The area under the ROC curve for PS (22:0/22:1(11Z)) and C17-Sphinganine were determined to be 0.976 and 0.913, respectively. These metabolites may potentially serve as diagnostic markers for sepsis. Additionally, the pathogenesis of sepsis is associated with mTOR signaling, NF-κB signaling pathway, calcium signaling, calcium transport, and tRNA charging pathway.

**Conclusion:**

The identification of differential expression of these metabolites in sepsis serum samples could aid in the timely diagnosis and intervention of sepsis, as well as enhance our understanding of its pathogenesis.

**Supplementary Information:**

The online version contains supplementary material available at 10.1186/s12920-023-01666-w.

## Background

Sepsis is a clinical syndrome characterized by physiological and organ dysfunction resulting from an impaired inflammatory response to infection [1]. The worldwide prevalence of sepsis and severe sepsis is reported to be 4370 per million and 2700 per million, respectively, with an annual mortality rate of 5.3 million [2]. It is noteworthy that sepsis is the leading cause of death among hospitalized patients on a global scale. Timely diagnosis is imperative for the effective management of sepsis, as initiating prompt treatment is crucial in reducing mortality associated with severe sepsis. The timely initiation of specific and appropriate treatment has the potential to enhance the prognosis of sepsis through early diagnosis and patient stratification. Nevertheless, the determination of sepsis severity during the initial stages can pose challenges [3]. Currently, there is a lack of sepsis biomarkers that exhibit sufficient specificity or sensitivity [4]. Employing a combination of multiple biomarkers proves to be more effective than relying on a single molecule, thereby offering potential for optimizing the diagnosis and treatment of sepsis.

The application of liquid chromatography-mass spectrometry (LCMS) in metabonomic analysis has the capability to concurrently detect a substantial number of potential novel biomarkers in sepsis patients. The utilization of this particular phenotype in subsequent studies holds promise as a valuable means for monitoring both the efficacy of therapeutic interventions and the progression of the disease [5].

In our study, the application of LCMS/MS-based metabonomics was employed to examine the metabolic profiles in serum samples obtained from both sepsis patients and a healthy control group. The primary objective was to identify potential metabolic biomarkers associated with sepsis. Additionally, bioinformatic methodologies were utilized to analyze the identified biomarkers, aiming to elucidate the underlying pathogenesis of sepsis and enable timely diagnosis and intervention based on this valuable information.

## Materials and methods

### Study design and study population

The study protocol was approved by the Ethics Committee of Ganzhou Hospital, Nanchang University. Informed consent was obtained from all participants. Inclusion criteria were: adult patients aged 48–82 diagnosed with sepsis based on the 2001 consensus criteria; no history of cardiopulmonary resuscitation (CPR), surgery, pregnancy, or chronic kidney disease. Exclusion criteria were: parenteral nutrition within 48 h of admission; preexisting diabetes, metabolic disorders, chronic liver disease, AIDS, or decreased polymorphonuclear cells. Healthy controls had no acute or chronic illnesses, hospitalizations, or medications in the preceding 6 months, and had normal physical and lab examinations.

Serum samples were collected from sepsis patients within 24 h of ICU admission before treatment, and from fasting healthy controls in the morning. Drawn blood was immediately placed on ice, centrifuged at 3500 rpm for 5 min after 1 h, then 100 µL serum was mixed with 300 µL methanol, vortexed for 2 min and centrifuged at 12,000 rpm for 15 min. The resulting 200 µL supernatant was used for LC-MS/MS detection.

### LC-MS/MS data acquisition

In this experiment, chromatographic separation was performed using an ACQUITY UPLC HSS T3 column (2.1 × 100 mm I.D., 1.8 μm, Waters, USA). The column was maintained at a temperature of 35 °C, and the mobile phases consisted of water (Phase A) and acetonitrile (Phase B) with the addition of 0.1% formic acid. The gradient program involved the application of a 5% solution of Phase B for the first 3 min, followed by a linear increase from 5 to 95% solution B over the next 12 min (3–15 min), and then maintaining the 95% solution B for an additional 2 min (15–17 min). A 5-minute post-treatment period was conducted with a flow rate of 350 µL/min. The sample injection volume was approximately 2 µL.

The data in this study were collected using Agilent 1290 LC and 6538 Q-TOF mass spectrometers, employing electrospray ionization in both positive and negative ionization modes. The drying gas N2 was delivered at a rate of 9 L/min, while the temperature was maintained at 360 °C. The nebulizer pressure was set at 39 psi, and the capillary voltage was adjusted to 4000 V and 3900 V for positive and negative ionization modes, respectively. The scan range for the mass spectra was set from 50 to 1000 m/z. To ensure accuracy and reproducibility, a reference solution was utilized for real-time correction of the mass spectra, with the lock mass (m/z 922.009798, 121.050873) serving as a reference point.

### Statistical analysis

The mass data matrix was generated using the Agilent Mass Hunter Quant B.07.00 software (Agilent Technologies Inc., Santa Clara, CA, USA) and the XCMS package in the R platform. This matrix encompassed the m/z, retention time, and peak area values for all ions. The peak area of each metabolite in each sample was normalized by the sum of the total peak areas in that sample. Prior to conducting multivariate statistical analysis, the data were standardized to unit variance and centered using Simca-p software 13.0. The datasets underwent Principal Component Analysis (PCA) and Orthogonal Partial Least Squares Discriminant Analysis (OPLS-DA) analysis. To assess the risk of overfitting, the OPLS-DA models were subjected to a permutation test. Additionally, potential biomarkers were identified by comparing the fragment ion and molecular weight information using metabolomic databases (METLIN, https://metlin.scripps.edu/; HMDB, http://www.hmdb.ca/), specifically focusing on ions with a Variable Importance in Projection (VIP) score greater than 1 and a p-value less than 0.05. Bioinformatic analysis was conducted to perform multiple testing of univariate data analysis. Furthermore, the retention time was compared to a reference to validate the metabolite structure. Receiver operating characteristic (ROC) curve analyses were employed to evaluate the diagnostic performance of the differential metabolites using the Biomarker analysis module implemented in Metaboanalyst 5.0 [6] by upload the expression of metabolites (http://www.metaboanalyst.ca). AUC was calculated from ROC analysis.

### Bioinformatics analysis

The changed metabolites were entered into MetaboAnalyst 5.0 using their HMDB IDs for pathway analysis. Information from Kyoto encyclopaedia of genes and genomes (KEGG) [7–9]and human metabolome database (HMDB) for metabolic pathway analysis was used. Pathway analysis with metabolite expression data was evaluated for statistical significance using the hypergeometric test and pathway topology was analyzed based on the relative-betweenness centrality. A pathway with an impact factor > 0.1 was considered an important pathway. Functional and network analysis was done using Ingenuity Pathway Analysis (IPA; Ingenuity Systems, www.ingenuity.com, Mountain View, CA) by uploaded the information of the changed metabolites, such as the HMMD ID, fold change and P value. Ingenuity pathway analysis is a web-based software application that enables to analyze and integrate data derived from metabolites into biological networks and pathways. All Ingenuity products leverage the Ingenuity Knowledge Base, which houses biological and chemical relationships extracted from the scientific literature.

## Results

Clinical information of patients.

This study included a total of sixty participants from the Department of Emergency, the Affiliated Ganzhou Hospital of Nanchang University. Among these participants, thirty were classified as normal controls while the remaining thirty were diagnosed with sepsis. The general characteristics of the study participants are presented in Table 1. The findings indicate that the sepsis group exhibited significantly higher scores in body temperature, C-reactive protein (CRP), procalcitonin (PCT), blood urea nitrogen (BUN), SOFA, and APACHE II compared to the control group (p < 0.05).

**Table 1:** Demographics of the enrolled patients in this metabolomics pilot study

ID	Characteristics	Control, n = 30	Sepsis, n = 30	p
1	Age (years)	58 ± 10	60 ± 15	0.4
2	Gender	15/15	15/15	0.3
3	Temperature (°C)	37.1 ± 0.7	37.9 ± 0.9	0.01
4	WBC counts (×10^9^/L)	7.26 ± 1.32	14.05 ± 7.36	0.02
5	Serum CRP (mg/L)	2.32 ± 1.21	11.54 ± 5.78	0.001
6	Serum PCT (ng/mL)	0.27 (0.2–0.54)	2.28 (0.6–7.12)	0.005
7	BUN (mmol/L)	5.02 ± 3.18	8.01 ± 3.21	0.005
8	Serum Creatinine (mmol/dL)	63.5 (39.5–75.3)	79.5 (55.5–140)	0.001
9	APACHE II score	11 ± 9	22 ± 7	
10	SOFA score		9 ± 4	
11	Pulmonary			
12	Urinary		11 (31.4)	
13	Bacteremia		9 (25.7)	
14	Gram-positive bacteria		17 (48.6)	
15	Gram-negative bacteria		34 (97.1)	
16	Fungi		22 (62.90)	

CRP, C-reactive protein; PCT, procalcitonin; BUN, blood urea nitrogen; SOFA, sequential organ failure assessment

### Multivariate analysis

**Fig. 1:**
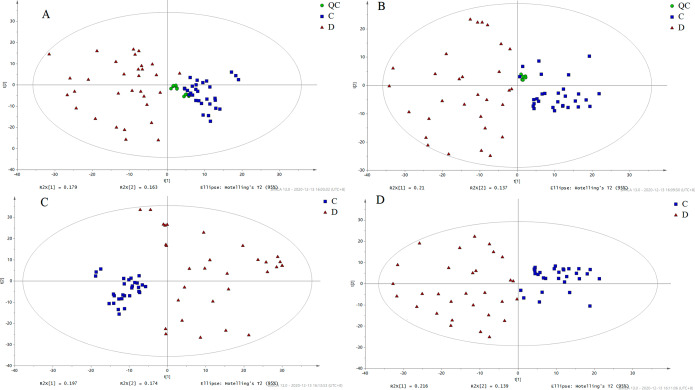
The score plot of PCA analysis. **(A)** POS, QC sample, sepsis group (labeled as D) and the control group (labeled as C); **(B)** NEG, QC sample, sepsis group (labeled as D) and control group (labeled as C); **(C)** POS, sepsis group (labeled as D) and control group (labeled as C); **(D)** NEG, sepsis group (labeled as D) and control group (labeled as C)

**Fig. 2:**
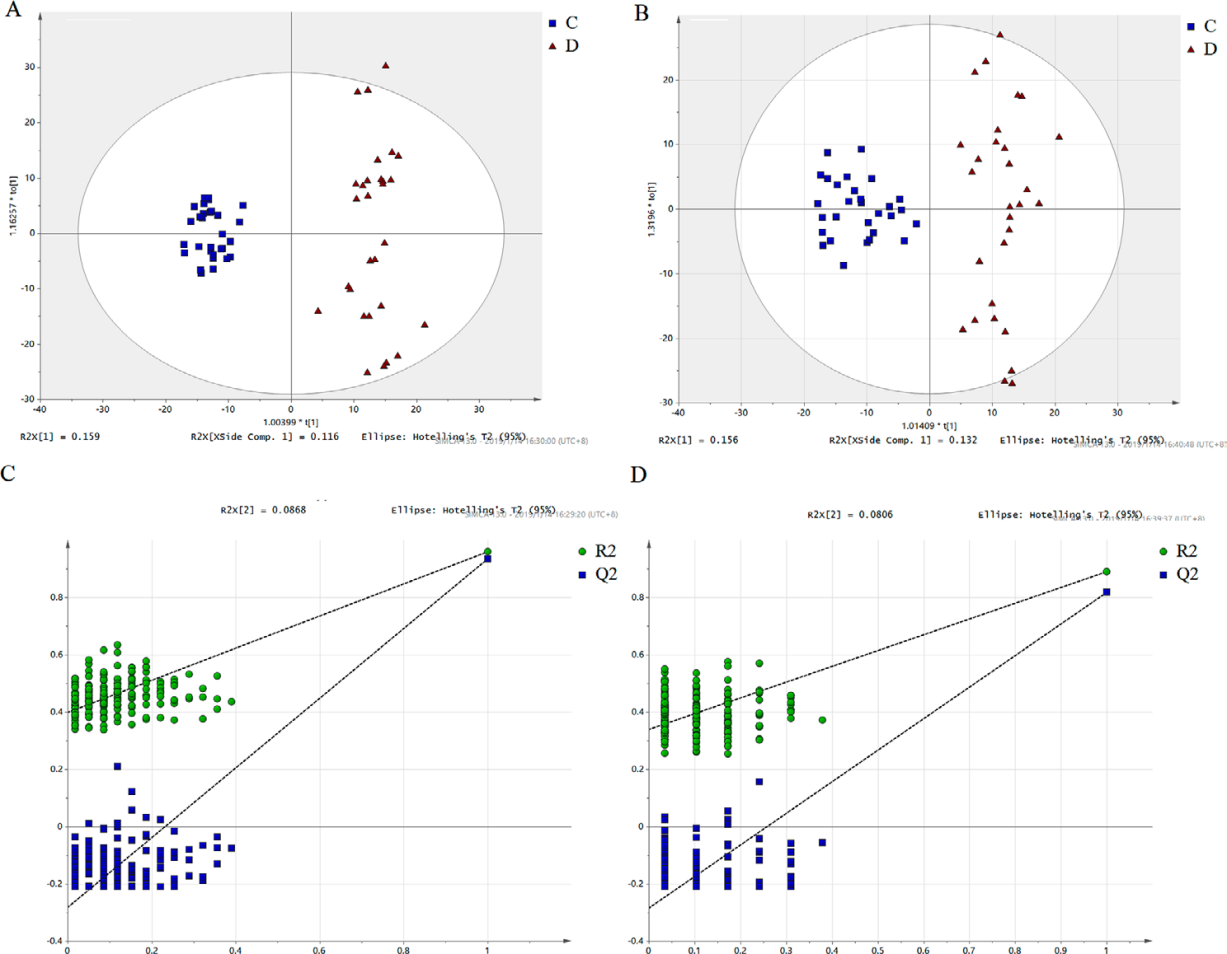
The score of OPLS-DA analysis. **(A)** POS, sepsis group (labeled as D) and control group (labeled as C) (R2X = 0.275, R2Y = 0.959, Q2 = 0.935 in the positive mode); **(B)** NEG, sepsis group (labeled as D) and control group (labeled as C) (R2X = 0.288, R2Y = 0.89, Q2 = 0.829 in the negative mode); **C**, The permutation test for the OPLS-DA model(POS, Intercepts: R2(0.0,0.408),Q2=(0.0,-0.284); **D**, The permutation test for the OPLS-DAmodel(neg, Intercepts: R2(0.0,0.341), Q2=(0.0,-0.28)

**Fig. 3:**
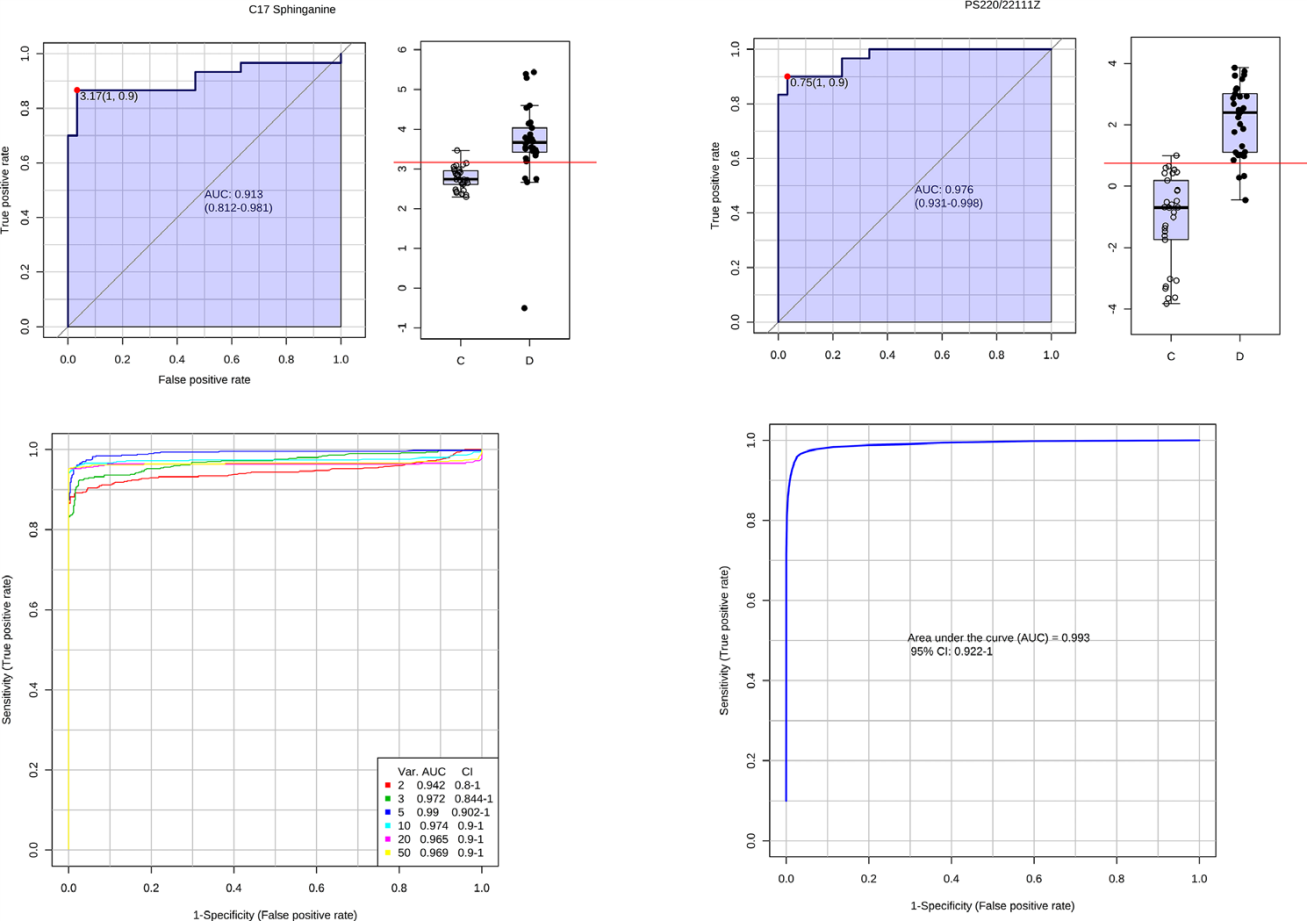
Receiver operating characteristic (ROC) curves for the metabolites between the control (*n* = 30) and sepsis (*n* = 30) groups. AUC indicates area under the curve

**Fig. 4:**
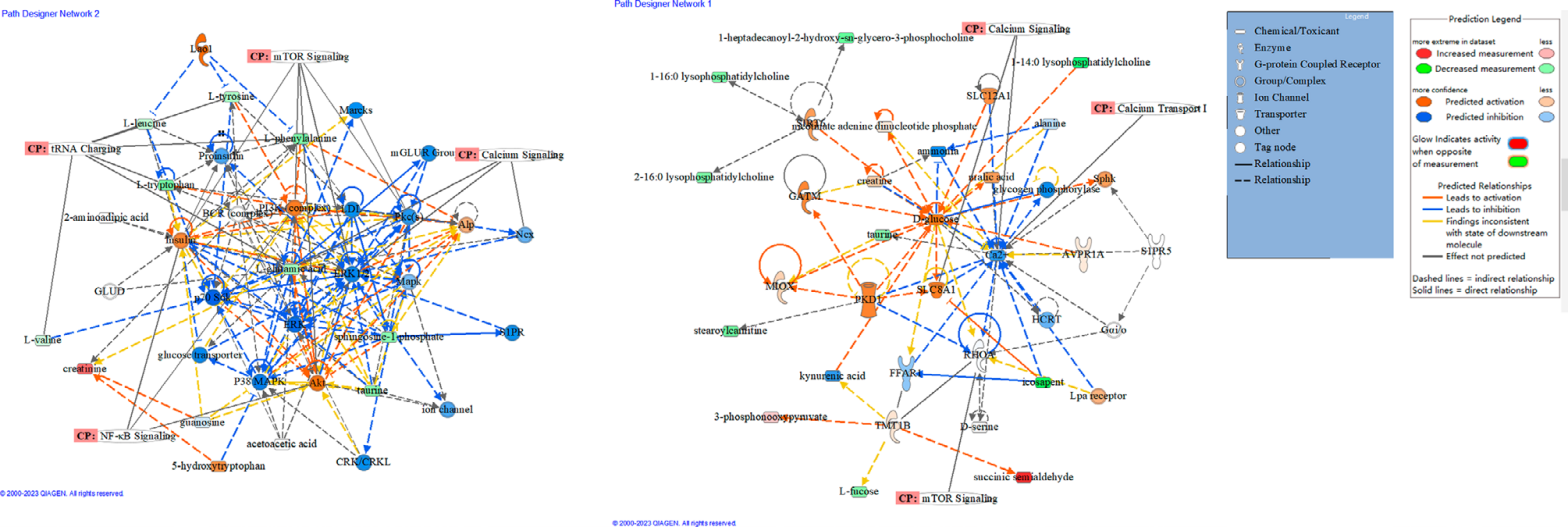
The network analysis of the differentially expressed metabolites in the sepsis group. CP represents the signal pathway; red nodes represent the increased metabolites in sepsis; and green nodes represent the decreased metabolites in sepsis. Black solid lines represent direct connections, and black dotted lines represent indirect associations

In Fig. 1, the PCA score plot displays the samples of sepsis and control. The positive model yielded two principal components with R2X = 0.57 and Q2 = 0.299, while the negative model also yielded two principal components with R2X = 0.568 and Q2 = 0.33. The tight clustering of the QC samples indicates the stability of the instrument. A variety of potential biomarkers from diverse groups were subjected to screening using OPLS-DA models. The OPLS-DA score plot (Fig. 2A, B) clearly demonstrates the ability to distinguish between the control group and the sepsis group. The OPLS-DA model generated a principal component and an orthogonal component. The positive model (R2X = 0.27, R2Y = 0.958, Q2 = 0.936) and negative model (R2X = 0.28, R2Y = 0.88, Q2 = 0.829) of the OPLS-DA exhibited favorable accuracy and predictability, as depicted in Figs. 2A and B and 417 features (VIP > 1.0) based on positive and 356 features (VIP > 1.0) based on negative modes were used for OPLSDA modes, as shown in Fig. S1. To mitigate the risk of overfitting the OPLS-DA model, cross-validation was performed using the default 7-fold procedure. Moreover, the permutation test outcomes of the OPLS-DA model indicate that the model has not suffered from overfitting, thereby suggesting its reliability (POS, intercepts: R2(0.0,0.408), Q2= (0.0, -0.284); NEG, intercepts: R2(0.0,0.341), Q2=(0.0,-0.28))(Fig. 2C, D). To identify the endogenous metabolites, a combination of high-resolution mass spectrometry and multivariate analysis was employed. Subsequently, we proceeded to perform MS/MS structure analysis on the ions that exhibited significance (VIP > 1 and p < 0.05) in the OPLS model. Subsequently, the Metlin database (http://metlin.scripps.edu) and HMDB database (http://www.hmdb.ca) were consulted to obtain the molecular mass data (m/z) and fragment ions of the aforementioned ions. Subsequently, the structure of the putative biomarkers was verified by comparing the retention time and MS/MS fragment with the reference standards available in our laboratory. Ultimately, a total of 59 metabolites were identified as Sepsis-related biomarkers (as presented in Table 2). Heatmaps and volcano plots and were used to show the differential metabolites between these two groups of sepsis group and control group as shown in Fig. S2. Bioinformatic analysis was conducted to account for multiple testing in the univariate data analysis, and the adjusted P value is displayed in Table 2. A significant decrease was observed in the levels of most PCs, LysoPCs, PSs, and LysoPEs in the sepsis group compared to the control group as shown in Fig. S3. Conversely, the levels of Creatinine, C17 Sphinganine, and PS (22:0/22:1(11Z)) were found to be increased in sepsis. The ROC curve analysis was performed on the 59 metabolites that exhibited marked changes (Fig. 3). The area under the ROC curve for PS (22:0/22:1(11Z)) and C17-Sphinganine were determined to be 0.976 and 0.913, respectively. These findings suggest that PS (22:0/22:1(11Z)) and C17-Sphinganine may serve as potential diagnostic markers for sepsis. The network analysis revealed that the NF-kappa B signaling pathway, tRNA charging, mTOR signaling pathway, calcium signaling pathway, and calcium transport were significantly associated with the observed alterations in metabolites. These findings suggest a potential link between these pathways and the pathogenesis of sepsis, as depicted in Fig. 4. Furthermore, the detailed metabolomic pathway analysis can be found in Table S1 of the supplementary materials. Kaplan Meier’s analysis showed that the CRP, PCT and APACHEII for the diagnosis of sepsis as shown in Fig S4. According to p values of Kaplan Meier’s analysis of CRP, PCT, and APACHE I they are not good predictors to separate sepsis from control.

**Table 2:** Metabolite differences that were identified between the different groups

ID	MZ		name	RT	VIP	Adust p value	FC(sepsis/control)
HMDB0248231	219.113		1-Methyl-L-tryptophan	8.86	1.61	2.03E-04	1.28
HMDB0000933	229.1373		2Z-Dodecenedioic acid	4.90	1.05	4.18E-03	0.66
HMDB0001494	140.9958		Acetyl phosphate	16.34	2.34	7.62E-06	1.21
LMSP01040003	288.2901		C17 Sphinganine	7.39	1.43	1.77E-03	1.16
LMSP01050004	416.2528		C19 Sphingosine-1-phosphate	4.90	1.30	6.62E-05	0.59
HMDB0006510	494.3245		Cervonyl carnitine	8.58	1.16	2.98E-04	0.55
HMDB0002586	473.2649		Chenodeoxycholic acid 3-sulfate	8.46	1.01	2.90E-02	1.13
HMDB0000562	114.0663		Creatinine	0.74	1.20	7.05E-04	1.75
HMDB0242127	393.2863		Docosanedioic acid	8.37	1.45	2.64E-02	1.09
HMDB0001999	303.2319		Eicosapentaenoic Acid	9.78	1.08	8.14E-04	0.36
HMDB0000174	187.0542		Fucose	0.78	1.23	1.39E-04	0.59
HMDB0000148	148.0582		Glutamate	0.73	1.09	5.87E-04	0.65
HMDB0252892	285.9789		Glyphosine	0.70	1.40	2.11E-05	0.71
LMFA01170040	425.3621		Hexacosanedioic acid	12.75	1.36	3.19E-04	0.59
HMDB0000687	130.0847		Leucine	1.39	1.36	1.26E-03	0.76
HMDB0000159	188.0708		L-Phenylalanine	4.23	1.69	3.04E-07	0.58
HMDB0010381	480.3082		LysoPC(15:0)	9.30	1.17	2.07E-03	0.67
HMDB0010393	546.3556		LysoPC(20:3(5Z,8Z,11Z))	9.26	1.26	1.12E-04	0.52
HMDB0011475	502.2931		LysoPE(0:0/18:1(11Z))	8.78	1.11	5.18E-04	0.53
HMDB0011482	508.3396		LysoPE(0:0/20:1(11Z))	9.04	1.29	6.62E-05	0.53
HMDB0011483	504.3081		LysoPE(0:0/20:2(11Z,14Z))	8.90	1.17	1.77E-03	0.63
HMDB0011515	526.293		LysoPE(20:3(5Z,8Z,11Z)/0:0)	8.75	1.21	1.84E-04	0.49
LMGP10010933	587.3788		PA(14:0/12:0)	4.76	1.38	2.11E-05	0.55
LMGP01050012	468.3089	PC(14:0/0:0)		8.30	1.28	8.38E-05	0.42
HMDB0010382	496.3407		PC(16:0/0:0)	9.30	1.18	2.39E-04	0.58
HMDB0012108	510.3558		PC(17:0/0:0)	9.92	1.11	5.17E-04	0.53
LMGP01050030	522.3563		PC(18:1(9E)/0:0)	9.58	1.12	5.00E-04	0.55
LMGP01050034	520.3406		PC(18:2(2E,4E)/0:0)	8.89	1.15	3.45E-04	0.48
LMGP01050068	398.2418		PC(9:0/0:0)	8.37	1.38	3.80E-02	1.08
LMGP02010102	490.268		PE(8:0/8:0)	4.73	1.39	2.07E-05	0.56
LMGP04050031	527.3212		PG(19:0/0:0)	4.96	1.30	6.62E-05	0.59
LMGP04010030	441.2013		PG(6:0/6:0)	12.74	1.55	2.01E-05	0.78
LMGP04030093	835.5961		PG(P-20:0/22:6(4Z,7Z,10Z,13Z,16Z,19Z))	16.30	1.49	5.36E-06	0.33
HMDB0001024	184.9858		Phosphohydroxypyruvic acid	16.34	2.39	3.19E-06	1.23
HMDB0240262	601.3363		PI(18:0/0:0)	4.84	1.29	6.62E-05	0.58
LMGP06050027	649.305		PI(19:0/0:0)	8.90	1.52	1.03E-04	0.63
LMGP06010442	923.6567		PI(19:0/21:0)	10.45	1.11	1.26E-03	2.58
LMGP06010443	937.6551		PI(19:0/22:0)	12.09	1.39	2.11E-05	0.17
LMGP06070002	583.3347		PI(P-18:0/0:0)	9.58	1.34	3.84E-04	0.59
LMGP06030075	913.6358		PI(P-20:0/19:1(9Z))	10.38	1.57	4.46E-06	3.24
LMGP03010027	658.3315		PS(12:0/12:0)	9.25	1.51	8.38E-05	0.59
LMGP03010001	638.3899		PS(12:0/13:0)	5.02	1.31	6.18E-05	0.58
LMGP03050005	478.3294		PS(13:0/0:0)	9.30	1.07	8.78E-04	0.67
LMGP03050030	534.2959		PS(17:0/0:0)	9.30	1.16	3.05E-04	0.67
LMGP03050006	526.3045		PS(18:0/0:0)	9.30	1.22	1.53E-04	0.59
LMGP03050028	538.3135		PS(19:0/0:0)	8.58	1.29	5.69E-04	0.60
LMGP03050020	552.33		PS(20:1(11Z)/0:0)	5.44	1.42	2.07E-05	0.54
LMGP03050026	566.3454		PS(21:0/0:0)	9.58	1.30	5.53E-04	0.61
LMGP03010722	924.6452		PS(22:0/22:1(11Z))	10.63	1.67	1.93E-06	3.66
LMGP03020054	800.5824		PS(O-20:0/18:3(6Z,9Z,12Z))	16.45	1.56	2.30E-06	0.38
HMDB0000277	380.2564		Sphingosine-1-phosphate	8.20	1.91	1.76E-09	0.60
HMDB0000848	428.3736		Stearoylcarnitine	9.30	1.38	2.11E-05	0.55
HMDB0001259	101.0244		Succinic acid semialdehyde	1.35	1.23	6.73E-04	2.29
HMDB0000251	124.0074		Taurine	0.73	1.45	1.60E-04	0.60
HMDB0002581	618.2472		Taurocholic acid 3-sulfate	9.30	1.60	1.93E-06	0.63
HMDB0010562	1045.711		TG(22:6(4Z,7Z,10Z,13Z,16Z,19Z)/22:6(4Z,7Z,10Z,13Z,16Z,19Z)/22:6(4Z,7Z,10Z,13Z,16Z,19Z))	10.13	1.52	3.41E-06	0.20
HMDB0000929	203.0823		Tryptophan	4.23	1.84	2.30E-06	0.56
HMDB0000158	180.0664		Tyrosine	1.31	1.50	2.25E-04	0.69
HMDB0000883	118.0862		Valine	0.81	1.08	1.25E-03	0.84

MZ, mass-to-charge ratio;VIP, variable importance in projection;FDR_bh, ajust p value; .FC, fold chage of sepsis/control

## Discussion

Recent advancements in systems biology and metabonomics have significantly enhanced the value of metabolomics in the clinical management of critically ill patients [10]. Previous metabolomic studies have revealed remarkable alterations in metabolites that could facilitate the diagnosis of infections and assessment of prognoses [1].

In this study, we screened serum biomarkers of sepsis utilizing LC-MS/MS. Compared to healthy controls, sepsis patients exhibited markedly differentiated metabolite profiles (Fig. 1). Bioinformatic analysis enabled the identification of 59 differential metabolites with potential as biomarkers for sepsis. Notably, 34 of these were phospholipids and 4 were fatty acids, diverging substantially from prior focuses on amino acids [5]. As anticipated, sepsis patients displayed decreased levels of glutamate, leucine, phenylalanine, tryptophan and tyrosine [5]. Serum dimethylamine concentrations were found to be elevated in septic shock patients with acute kidney injury by targeted liquid chromatography-tandem mass spectrometry [11].

Our findings align with previous reports linking imbalanced amino acid profiles with higher mortality in sepsis patients [12]. Phenylalanine and tryptophan may serve as indicators of susceptibility to adverse outcomes as essential amino acids. While elevated phenylalanine signified severe metabolic disruption in one cohort, another study observed decreased essential amino acids in sepsis [13].Highlighting heterogeneous stress responses among patients. The intricate mechanisms regulating amino acid fluctuations remain multifaceted and interrelated.

Uniquely, over 50% of the differential metabolites were phospholipids, encompassing phosphatidylcholines (PCs), phosphatidylethanolamines (PEs), phosphatidylinositols(PIs), and phosphatidylserines(PSs), which have not been previously reported. Previous studies have indicated that the elevations in HDL-POVPC during sepsis were significantly associated with 28-day mortality, indicating prognostic utility, suggesting their potential as prognostic markers. The findings highlight the importance of HDL-POVPC in sepsis progression and its potential as a predictor of poor outcomes [14]. To evaluate the diagnostic potential of these metabolic biomarkers, receiver operating characteristic (ROC) curve analysis was performed. Results revealed phosphatidylserine PS(22:0/22:1(11Z)) and C17-sphinganine as having the highest sensitivity among the candidate sepsis indicators.Phosphatidylserine PS(22:0/22:1(11Z)) is a glycerophospholipid possessing three ionizable groups, which exhibits a wide distribution across animals, plants, and microorganisms [4]. Phosphatidylserine PS(22:0/22:1(11Z)) is an integral membrane phospholipid located primarily on the outer leaflet of cell membranes, where it maintains membrane structural stability [15]. C17-sphinganine is a type of sphinganine lipid essential for cell membrane composition [16]. Studies in animal sepsis models have shown decreased PS(22:0/22:1(11Z)) and C17-sphinganine levels in hepatic and muscular tissues [17, 18]. These changes may result from inflammatory factors, oxidative stress, and phospholipase activation during sepsis, compromising membrane integrity and impairing cell function. However, some studies report upregulated plasma PS(22:0/22:1(11Z)) levels in sepsis patients [19]. Elevated PS could increase membrane fluidity, disrupt membrane proteins, and promote coagulation and inflammation. In summary, altered expression of PS(22:0/22:1(11Z)) and sphinganine C17 are closely implicated in sepsis pathogenesis through various potential mechanisms, though the precise molecular pathways remain to be elucidated. These results suggest PS(22:0/22:1(11Z)) and C17-sphinganine may serve as a specific sepsis biomarker warranting further investigation.

Furthermore, our study revealed the presence of C19 sphingosine-1-phosphate, a previously unreported metabolite, in sepsis patients. This novel finding has not been documented in other metabolomic studies of sepsis. Notably, the analog sphingosine-1-phosphate (S1P) has been recognized as a potential therapeutic agent for sepsis [20]. S1P is a critical immunomodulator regulating immune function and organ protection. Carrier molecules transporting S1P play a crucial role in modulating its effects, and their decrease in septic shock may contribute to disease severity [21]. In animal models of sepsis-induced lung injury, S1P demonstrated efficacy in stabilizing endothelia and improving survival. Interestingly, we observed decreases in both S1P and C19 sphingosine-1-phosphate in sepsis. The signaling lipid S1P participates in various pathophysiological processes including modulating vascular permeability, promoting inflammation, and influencing coagulation [22–24]. S1P lyase activity is higher in tissues while blood and lymph exhibit greater S1P levels. Preclinical studies have revealed associations between sepsis and reduced S1P levels in serum/plasma, suggesting S1P may be a marker of sepsis severity and onset [25]. Augmenting S1P levels could potentially improve outcomes in sepsis, warranting studies on modulating S1P activity via endothelial receptors.

## Conclusion

In conclusion, PS (22:0/22:1(11Z)) and C17-Sphinganine exhibit potential as distinctive indicators of sepsis in forthcoming research. To substantiate this discovery and delve into the pathophysiology of sepsis, additional prospective studies are imperative. One limitation of this study is the relatively small sample size, highlighting the need for further validation of the identified sepsis biomarkers using larger sample sizes and multicenter data to enhance the reliability and generalizability of the findings.

### Electronic supplementary material

Below is the link to the electronic supplementary material.

Supplementary Material 1

## Data Availability

The author (Xiaoliu Xiao) will provide the raw data supporting the conclusions of this article if someone wants to request the data from this study.
