# Supplementary material for: Serum metabolite profiling reveals metabolic characteristics of sepsis patients using LC/MS-based metabolic profiles: a cross-sectional study
Source: BMC Med Genomics. 2023 Sep 26;16:224. doi: 10.1186/s12920-023-01666-w (PMC10521453; doi:10.1186/s12920-023-01666-w)
Supplement: Supplementary file 1 — Supplementary Material 1 [file 12920_2023_1666_MOESM1_ESM.pdf]

Supplemental Material

Serum metabolite profiling reveals metabolic characteristics of sepsis patients using LC/MS-based metabolic profiles: A cross-sectional study

Jinliang Peng[1], Chongrong Qiu[1], Jun Zhang[1], Xiaoliu Xiao[1,*]

1 Department of Emergency, The Affiliated Ganzhou Hospital of Nanchang university, Jiangxi Province, 341000, China.

***Corresponding authors**

Xiaoliu Xiao, Department of Emergency, The Affiliated Ganzhou Hospital of Nanchang university, Jiangxi Province, 341000, China.

E-mail: 3065813189@qq.com;.

Supplemental Material Table S1. The pathway analysis of changed metabolites in the serum of sepsis patients compared with the control group.

Pathway name	Total	Expected	Hits	Raw p	Holm adjust	FDR	Impact
Aminoacyl-tRNA biosynthesis	48	0.43	6	1.60E-06	0.00	0.00	0.00
Phenylalanine, tyrosine and tryptophan biosynthesis	4	0.04	2	4.50E-04	0.04	0.02	1.00
Valine, leucine and isoleucine biosynthesis	8	0.07	2	2.06E-03	0.17	0.06	0.00
Phenylalanine metabolism	10	0.09	2	3.27E-03	0.27	0.07	0.36
Butanoate metabolism	15	0.14	2	7.44E-03	0.60	0.13	0.03
Alanine, aspartate and glutamate metabolism	28	0.25	2	2.51E-02	1.00	0.35	0.25
Valine, leucine and isoleucine degradation	40	0.36	2	4.86E-02	1.00	0.50	0.00
Nitrogen metabolism	6	0.05	1	5.31E-02	1.00	0.50	0.00
D-Glutamine and D-glutamate metabolism	6	0.05	1	5.31E-02	1.00	0.50	0.50
Taurine and hypotaurine metabolism	8	0.07	1	7.02E-02	1.00	0.59	0.43
Ubiquinone and other terpenoid-quinone biosynthesis	9	0.08	1	7.86E-02	1.00	0.60	0.00
Arginine biosynthesis	14	0.13	1	1.20E-01	1.00	0.84	0.12
Histidine metabolism	16	0.14	1	1.36E-01	1.00	0.88	0.00
Pantothenate and CoA biosynthesis	19	0.17	1	1.59E-01	1.00	0.93	0.00
Fructose and mannose metabolism	20	0.18	1	1.67E-01	1.00	0.93	0.00
Pyruvate metabolism	22	0.20	1	1.82E-01	1.00	0.96	0.00
Glutathione metabolism	28	0.25	1	2.26E-01	1.00	0.99	0.02
Porphyrin and chlorophyll metabolism	30	0.27	1	2.40E-01	1.00	0.99	0.00
Glyoxylate and dicarboxylate metabolism	32	0.29	1	2.54E-01	1.00	0.99	0.00
Cysteine and methionine metabolism	33	0.30	1	2.61E-01	1.00	0.99	0.00
Glycine, serine and threonine metabolism	33	0.30	1	2.61E-01	1.00	0.99	0.02
Biosynthesis of unsaturated fatty acids	36	0.33	1	2.81E-01	1.00	0.99	0.00
Glycerophospholipid metabolism	36	0.33	1	2.81E-01	1.00	0.99	0.02
Amino sugar and nucleotide sugar metabolism	37	0.33	1	2.88E-01	1.00	0.99	0.00

Arginine and proline metabolism	38	0.34	1	2.95E-01	1.00	0.99	0.09
Tryptophan metabolism	41	0.37	1	3.14E-01	1.00	1.00	0.14
Tyrosine metabolism	42	0.38	1	3.20E-01	1.00	1.00	0.14
Primary bile acid biosynthesis	46	0.42	1	3.45E-01	1.00	1.00	0.01

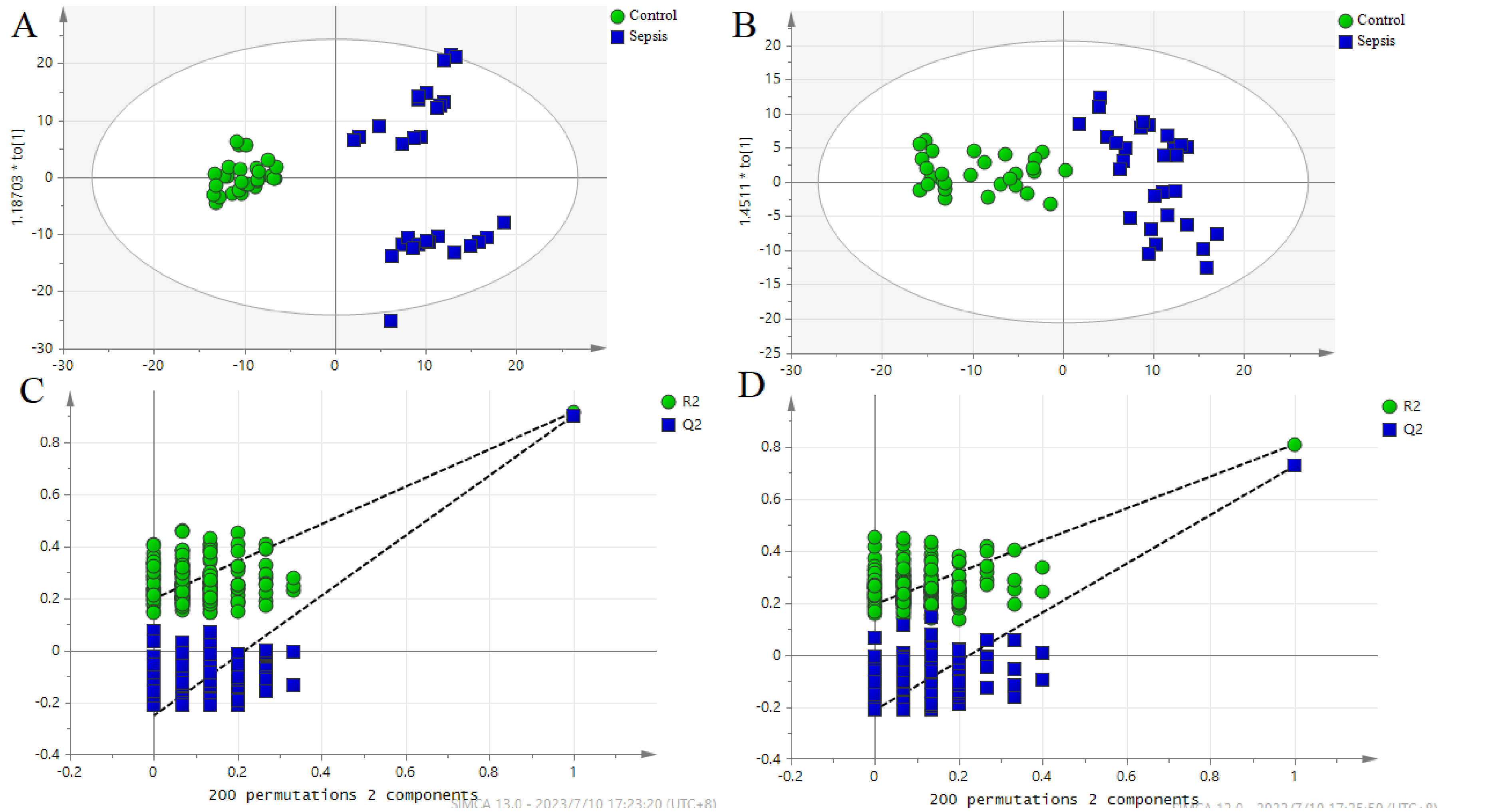

Fig. S1. The score of OPLS-DA analysis of metabolites with VIP > 1.2. A. POS, sepsis group and control group (R2X = 0.487, R2Y = 0.917, Q2

= 0.896 in the positive mode); B. NEG, sepsis group and control group (R2X = 0.506, R2Y = 0.81, Q2 = 0.743 in the negative mode); C, The permutation test for the OPLS-DA model(POS, Intercepts：R2(0.0,0.019),Q2=(0.0,-0.245); D,The permutation test for the OPLS-DAmodel(neg, Intercepts: R2(0.0,0.199), Q2=(0.0,-0.21).

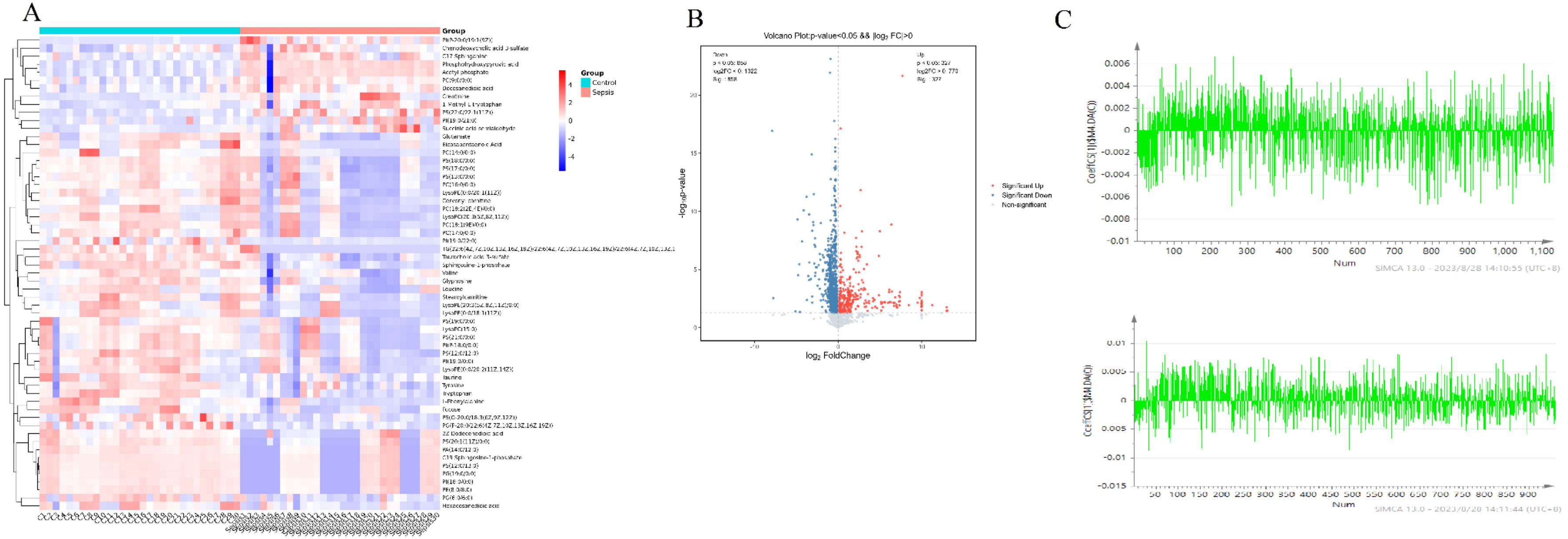

Fig. S2. Heatmaps, volcano plots and coefficient plot were used to show the differential metabolites between these two groups of sepsis group and control group.

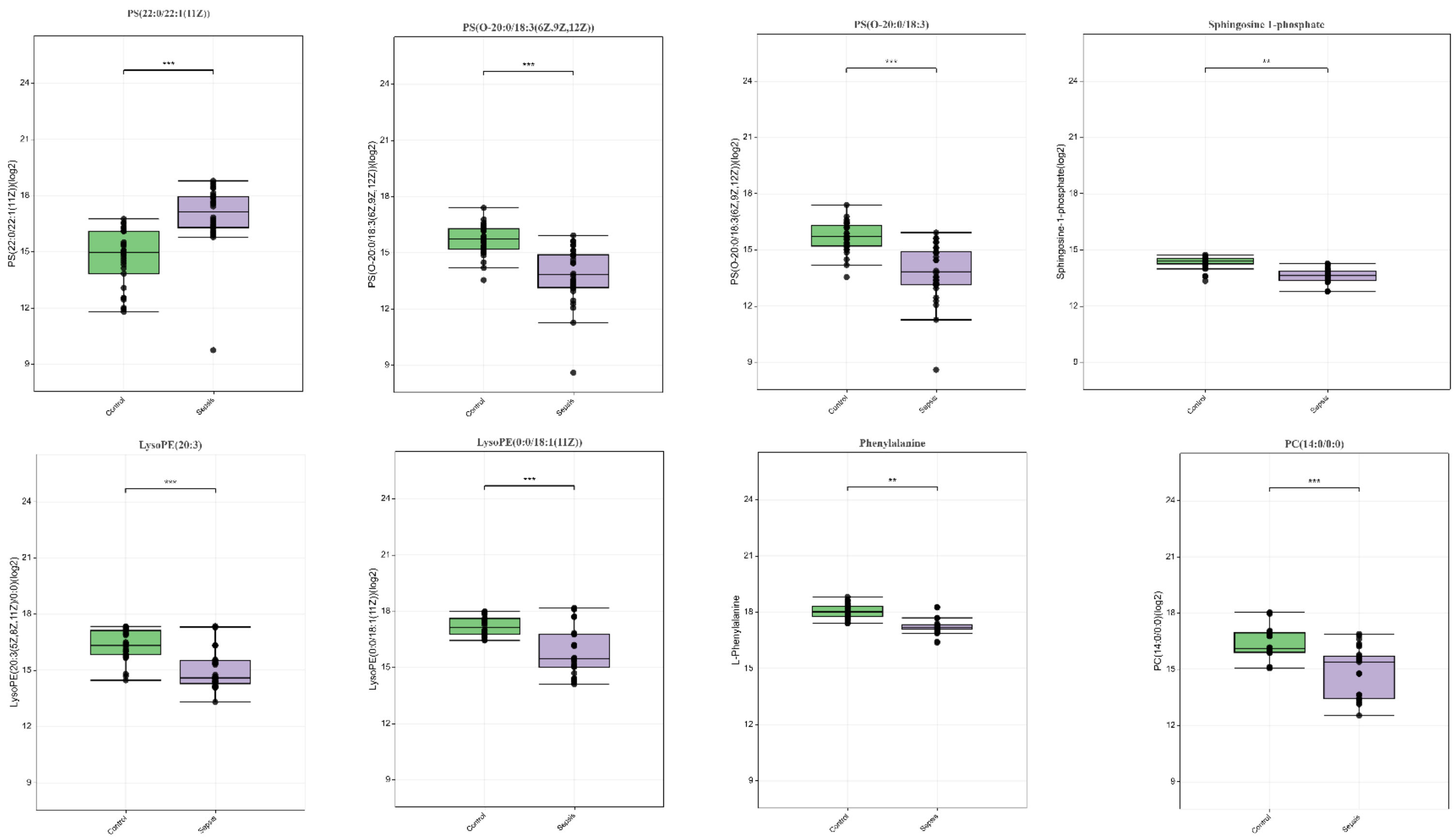

Fig. S3. Boxplots showing the difference in metabolite levels in serum samples for sepsis group and control group.

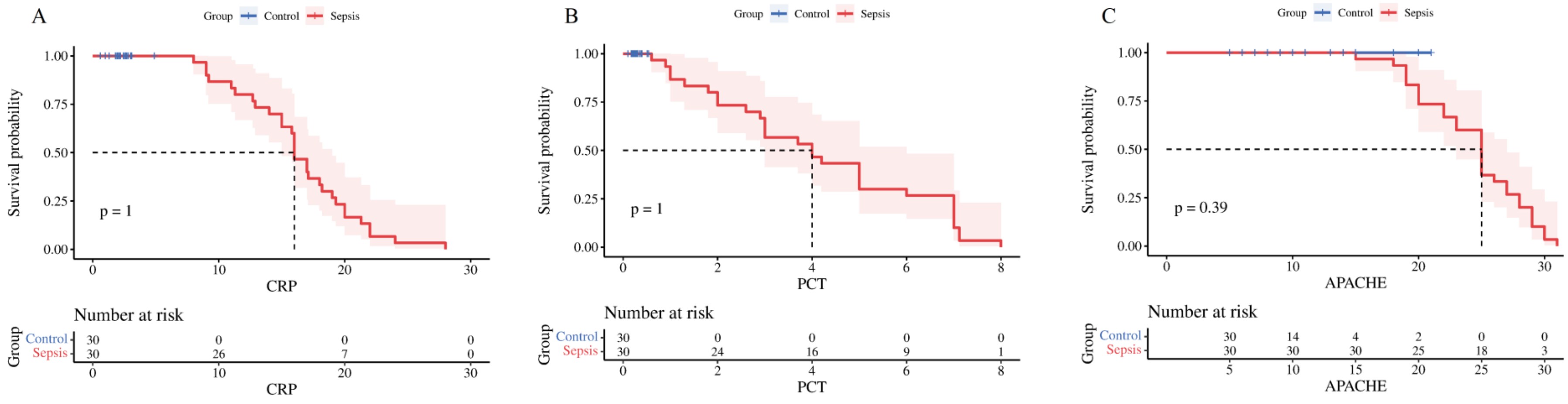

Fig. S4. Kaplan Meier's analysis showed that the CRP, PCT and APACHEII for the diagnosis of sepsis.
